# Mass Spectrometry-Based Methodologies for Targeted and Untargeted Identification of Protein Covalent Adducts (Adductomics): Current Status and Challenges

**DOI:** 10.3390/ht8020009

**Published:** 2019-04-23

**Authors:** João Nunes, Catarina Charneira, Judit Morello, João Rodrigues, Sofia A. Pereira, Alexandra M. M. Antunes

**Affiliations:** 1 Centro de Química Estrutural, Instituto Superior Técnico, ULisboa, 1049-001 Lisboa, Portugal; joaopcnunes@tecnico.ulisboa.pt (J.N.); catarina.charneira@tecnico.ulisboa.pt (C.C.); judit.morello@tecnico.ulisboa.pt (J.M.); 2 Clarify Analytical, Rua dos Mercadores 128A, 7000-872 Évora, Portugal; joao.rodrigues@clarifyanalytical.pt; 3 CEDOC, Chronic Diseases Research Centre, NOVA Medical School, Faculdade de Ciências Médicas, Universidade NOVA de Lisboa, 1169-006 Lisboa, Portugal; sofia.pereira@nms.unl.pt

**Keywords:** protein covalent adducts, adductomics, mass spectrometry

## Abstract

Protein covalent adducts formed upon exposure to reactive (mainly electrophilic) chemicals may lead to the development of a wide range of deleterious health outcomes. Therefore, the identification of protein covalent adducts constitutes a huge opportunity for a better understanding of events underlying diseases and for the development of biomarkers which may constitute effective tools for disease diagnosis/prognosis, for the application of personalized medicine approaches and for accurately assessing human exposure to chemical toxicants. The currently available mass spectrometry (MS)-based methodologies, are clearly the most suitable for the analysis of protein covalent modifications, providing accuracy, sensitivity, unbiased identification of the modified residue and conjugates along with quantitative information. However, despite the huge technological advances in MS instrumentation and bioinformatics tools, the identification of low abundant protein covalent adducts is still challenging. This review is aimed at summarizing the MS-based methodologies currently used for the identification of protein covalent adducts and the strategies developed to overcome the analytical challenges, involving not only sample pre-treatment procedures but also distinct MS and data analysis approaches.

## 1. Introduction

Protein covalent adducts are formed upon exposure to reactive chemical agents (mainly electrophiles) from internal or external exposures. Regardless of their nature, the possibility of identifying protein covalent adducts is of outmost importance due to their profound impact induced at both the molecular and cellular level, ultimately leading to the development of a wide range of deleterious health outcomes, including cancer, cardiovascular and autoimmune diseases [1,2,3,4,5,6].

Studies focused on the identification of protein covalent adducts are often classified as: 1) targeted, when focused on identifying covalent adducts formed upon exposure to a specific chemical agent; or 2) untargeted, aimed at comprehensively characterizing the totality of covalent conjugates bound to a specific nucleophilic residue of a protein. This last type of approach is generally referred to as adductomics and is used to characterize the exposome to reactive electrophiles. Nonetheless, the term “targeted adductomics” can also be used to characterize studies focused on comprehensively identifying the proteins (or the amino acid sites) that are targets for covalent adduction with a specific electrophile.

The ability to identify the nature of the covalent conjugate, along with its extent and the site of adduction within the protein structure is crucial towards a better understanding the molecular events underlying diseases, in general, and chemically-induced toxic events, in particular. Therefore, adductomics studies constitute a huge opportunity for the development of biomarkers that are anticipated to have a profound impact on human lives, as effective tools for disease diagnosis/prognosis and for accurately assessing human exposome [7,8]. Additionally, it can also guide regulatory agencies to make better decisions, thereby leading to minimization of chemically-induced adverse effects.

**Figure 1:**
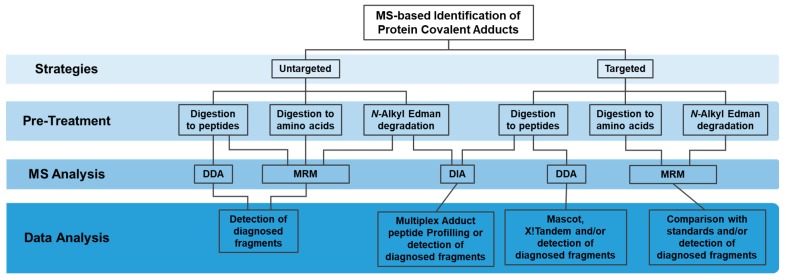
Strategies for the targeted and untargeted MS-based identification of protein covalent adducts.

The currently available High-Resolution Mass Spectrometry (HRMS)-based methodologies, in particular liquid chromatography coupled to tandem mass spectrometry (LC-MS/MS), enable accurate, sensitive and unbiased identification of the modified residue along with quantitative information. LC-MS/MS is clearly the most suitable method for the identification and quantification of covalent protein modifications. Three main mass spectrometry (MS)-based strategies have been used for the targeted and untargeted identification of protein covalent adducts formed with electrophiles of endogenous and exogenous exposure (Figure 1): 1) “bottom-up” strategies, involving protein digestion (mainly with trypsin) to peptides followed by MS analysis; 2) pronase digestion or acid hydrolysis of proteins to amino-acids followed by MS analysis; and 3) *N*-alkyl Edman degradation, involving the selective detachment of terminal adducted amino-acids, as hydantoins, upon reaction with isothiocyanates, followed by MS analysis. Nonetheless, the diversity of targets, the huge discrepancy of cellular proteins concentration and the low abundance of the modified protein when compared to the unmodified protein make the detection of protein adducts in a complex matrix a major challenge. Multiple strategies were, therefore, developed to overcome these analytical difficulties involving sample pre-treatment, MS analysis and data analysis procedures, which will be briefly summarized in this review.

## 2. Sample pre-treatment and adducts enrichment

A general procedure in the sample pre-treatment step of adductomics studies is the isolation of targeted proteins. Taking into consideration the low abundance of adducted proteins in vivo, the chances of identifying covalent adducts in a specific, pre-purified, protein are obviously simplified over the identification of adducts in a complex mixture of proteins. This strategy was successfully applied by our group for the identification of covalent adducts formed between an electrophilic furan metabolite and histone 2B, isolated from the livers of rats treated with tumorigenic doses of this hepatocarcinogen [9]. The basic character of histones, due to their high lysine content, allows the extraction of these nuclear proteins in an ice-cold acidic solution with minimal contamination from other proteins, which precipitate in this kind of environment. The subsequent trypsin digestion and LC-MS/MS analysis allowed the identification of a histone 2B peptide, containing Lys107 cross-linked with BDA (and electrophilic furan metabolite) and glutathione (GSH). This constituted the first in vivo report of covalent modification of histones by electrophilic metabolites of environmental and food contaminant carcinogens and the detection of this adduct prior to the identification of epigenetic changes suggests a role for this crosslink in the onset of furan-induced carcinogenicity. 

The possibility of identifying modifications of abundant blood proteins to comprehensively monitor the effect of chemical stress is recognized of major importance, since the abundance of proteins such as Human serum albumin (HSA) and hemoglobin (Hb) make complex sample cleanup procedures unnecessary. For instance, the isolation of HSA from blood is usually a simple pre-treatment procedure for the identification of covalent adducts formed with this serum protein. According to a recent review of Sabbioni and Turesky [10], the highest throughput to purified HSA is achieved by the fractional precipitation methods using ammonium sulfate without final precipitation of HSA; however, the highest levels of purity are achieved upon affinity purification followed by a low MWC filter. Nonetheless, the identification of HSA covalent adducts formed with an acyl glucuronide metabolite of the nonsteroidal anti-inflammatory drug diclofenac in human samples [11], involved the isolation of HSA from plasma by affinity chromatography. The purified protein was trypsin digested and prior to LC-MS/MS analysis, the tryptic peptides were subjected to ion exchange chromatography, which is argued to enhance substantially the sensitivity of the peptide analyses by LC-MS/MS. This work revealed for the first time the structure of the diclofenac-derived acyl conjugate in HSA and the formation of similar adducts with targeted proteins may constitute a possible cause of adverse reactions induced upon exposure to other acyl conjugates. 

Covalent adducts formed with Hb are also often used for biomonitoring proposes. An interesting approach for the identification of multiple covalent modifications in this abundant blood protein was presented by Oe’s group [12]. Following the Hb precipitation from hemolyzed red blood cells, by addition of ethyl acetate, these authors used multiple proteases (Trypsin, V8, and Asp-N) which yielded complimentary results upon MS analysis. Actually, the use of multiple proteases provided a broader picture of adducted peptides, allowing the identification of multiple covalent conjugates in distinct binding sites.

The purification of proteins is not always enough to allow adduct identification due to their low concentrations in vivo. Additionally, to fully understand cellular responses upon exposure to electrophilic (reactive) chemicals, it is important to determine the protein targets in an unbiased fashion. Therefore, several methodologies were developed to capture adducted peptides based on the reactivity of covalent conjugates, thereby improving the MS detectability of specific protein adducts in complex biological matrixes. 

**Figure 2:**
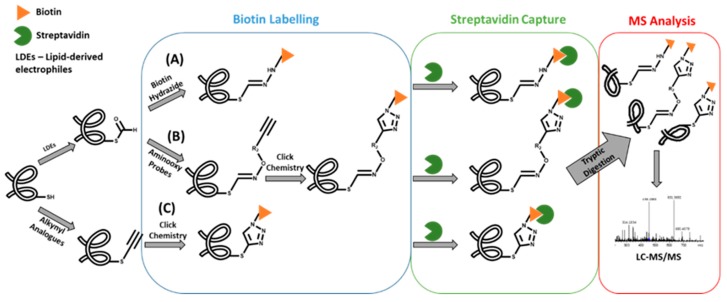
Strategies for the enrichment of protein alkynyl/aldehyde-containing covalent adducts.

One of the first examples of this strategy involved the use of biotin affinity probes to capture and, thereby, enrich protein and peptide adducts formed with lipid-derived electrophiles (LDEs) containing an aldehyde group [13,14,15]. This strategy was recently applied for the identification of acrolein protein adducts in human lung epithelial cells [16]. In fact, to date, three distinct approaches were developed for this strategy [17,18,19] (Figure 2): A) use of biotin hydrazide for covalently labeling the aldehyde group of the covalent conjugate followed by streptavidin capture for “fishing” the aldehyde-containing adducts; B) use of alkyne-functionalized aminooxy probes to react with aldehyde moieties of conjugates, allowing the subsequent functionalization with a cleavable azido-biotin tag (via the copper-catalyzed [3+2]-cycloaddition reaction of alkynes to yield triazoles - click chemistry) for subsequent enrichment via streptavidin catch [18]; and C) use of alkynyl analogs of lipid-derived [19] electrophiles, and post labeling of the resulting adducts with azidobiotin tags, by Click chemistry, which can be caught by streptavidin beads. A more recent application of this approach involved the use of a clickable probe of a neurotoxic metabolite of a drug candidate [20]. This allowed the identification of the general protein targets for covalent modification by this metabolite, thereby showing its ability to inhibit multiple mechanistically distinct enzyme classes involved in nervous system function. Similarly, a click reaction/MS-based strategy was used to identify the cellular protein targets of estrogen-derived quinones [21], which are electrophilic metabolites linked with the onset of diseases such as cancer. An estradiol derivative, containing an alkyne functional group, was used as the precursor probe together with a cleavable biotin hydrazide linker that enabled the click chemistry strategy. Subsequent LC-MS “bottom-up” proteomics approaches allowed the identification of protein targets in liver microsomes that catalyze the metabolism of estrogen-catechol (the metabolic precursors of estrogen quinones) and the detoxification of reactive oxygen species (ROS). Despite these useful applications, clickable probes as surrogates of electrophiles are not suitable for general exposome studies. Additionally, while the use of biotin probes has been amply applied for biomonitoring protein covalent adducts formed upon exposure to electrophilic species containing aldehyde (or alkynyl) moieties, its application to covalent conjugates with other functional groups is yet to be presented. In fact, the development of new chemical tools for the enrichment of covalent adducts bearing specific functional groups could be useful for further developments in protein adductomics studies. 

A methodology that is also employed for the enrichment of specific covalent adducts involves the use of polyclonal antibodies, which are developed to recognize specific adducts. This methodology was used by Meier et al. [22] for the detection of multiple adducts formed with quinone methide metabolites of the phenolic antioxidant butylated hydroxytoluene that were subsequently identified by LC-MS/MS analysis. A major limitation for the broad application of this strategy is the need to develop antibodies for each covalent conjugate.

One additional strategy for assessing the levels of covalent adducts formed with Hb and HSA is an indirect methodology that involves the acid or basic hydrolysis of the isolated adducted protein to release the electrophile-derived moiety [23,24,25,26] that is subsequently separated from the protein and quantified by suitable MS-based methods. However, this methodology can only be applied for the identification of very specific adducts that are prone to detachment from protein under mild acidic/basic conditions. 

Targeted and untargeted methodologies for the identification of covalent adducts formed in specific amino acids bearing a nucleophilic side chain (e.g., Cys, Lys, Hist or *N*-terminal Val of Hb) have also been useful tools for exposure studies. Namely, the pronase digestion of HSA to amino acids followed by targeted MS analysis of Lys adducts formed with 4,4’-methylenediphenyl diisocyanate (MDI), was used by Sabbioni et al. [27] to study the occupational exposure to this isocyanate in workers of a 4,4’-methylenedianiline factory. The digestion to amino acids, upon pronase digestion, was also used for the targeted quantification of Lys adducts formed with the food toxicant aflatoxin B1 [28].

With the aim of characterizing human exposome, MS-based untargeted approaches focused on the identification of multiple conjugates on specific residues of model proteins have emerged in the last decade. The most illustrative examples are those specifically focused on Cys34 adducts of HSA and *N*-terminal Val adducts of Hb that were recently extensively reviewed [29].

Rappaport´s group developed adductomics workflows focused on monitoring multiple covalent modifications occurring in the tryptic peptide containing Cys34 of HSA, which is a highly reactive and the sole Cys residue of HSA that is not involved in disulfide bridges. The use of thiol-affinity resins [30] and antibodies [31] was initially proposed for the enrichment of Cys34 adducts. However, more recent procedures do not use these pre-enrichment steps [32,33], the HSA is precipitated from serum with 60% methanol, and following trypsin digestion and prior to MS analysis an isotopically labeled peptide is added, as an internal standard, enabling corrections of retention time drifts and mass accuracy. Uchida´s group proposed a methodology based on the acid hydrolysis of proteins, such as low-density lipoproteins [34] and Hb [35], to amino acids for the comprehensive analysis of His and Lys adducts formed with lipid peroxidation products. The identification and quantification of adducts formed with *N*-terminal Val of Hb for biomonitoring proposes are based on a modified Edman procedure in which adducted *N*-terminal Val are specifically detached as hydantoins, upon reaction with an isothiocyanate. The demonstration of the in vivo bioactivation of the anti-HIV drugs nevirapine and abacavir to reactive metabolites [36,37,38] and the studies on the occupational and environmental exposures to ethylene oxide, acrylamide and acrylonitrile [39,40,41,42], are among the most significative applications of the targeted version of this methodology. Törnqvist’s group led the development of this approach and the shift to untargeted adductomics was possible with the use of fluorescein isothiocyanate (FITC), as the derivatizing agent [43]. Since FITC has a carboxylic acid functionality, a clean-up step involving anion exchange solid phase extraction column makes the removal FITC-by products possible prior to MS analysis, thereby enabling the application of this procedure for the identification of multiple covalent conjugates.

Recently, a methodology combining electrophoretic mobility band shift with MS was reported by Gesslbauer *et al*. [44], for the ex vivo identification of covalent adducts formed with multiple products of lipid peroxidation, which do not require the prior isolation or knowledge of target proteins. While the in vivo application of this technically relatively simple method has not yet been provided, it is potentially a methodology for application in the label-free analysis of protein covalent adducts.

## 3. MS Data Acquisition

MS-based “top-down” strategies, which consist on analyzing intact proteins, were proved to be powerful for the identification of labile epigenetic modifications (e.g., phosphorylations) [45]. However, taking into consideration that for instance HSA adducts occur at 0.1 mol% levels, or less, in vivo [9], the low levels of adducted proteins when compared to the non-adducted protein explains the failure of this strategy for adductomics studies. 

The general trend for protein adducts identification is to adopt the MS-based “bottom-up” workflows that became a standard in proteomics studies [46]. This strategy traditionally involves the chromatographic separation of tryptic peptides followed by MS analysis using a HRMS by data dependent analysis (DDA), where MS and MS/MS data are afforded in a single run. New generation HRMS spectrometers combine a good speed of scanning with high mass accuracy and this acquisition mode has, for instance, shown its applicability for the targeted identification of furan-derived histone adducts in a rat model [9] and for the untargeted detection and quantification of Cys34 HSA adducts in tryptic digests of human serum/plasma [32]. Turesky’s group have also successfully used DDA mode for the detection of HSA adducts formed upon exposure to heterocyclic aromatic amines [47] and aromatic amines [48]. Adducted peptides of trypsin and trypsin/chemotrypsin digests were characterized by ion trap mass spectrometry, employing isotopic data-dependent scanning. The methodology developed by Mathews et al. [49] for the identification of multiple organophosphorus nerve agent adducts formed with human butyrylcholinesterase (BChE) is one additional successful example of DDA application for covalent adduct detection. However, the inability to generate quality MS/MS spectra for precursor ions of low abundant protein adducts is the main reason preventing the general application of DDA mode for the identification of protein covalent adducts in vivo. In fact, by DDA only 10% of the detectable peptides are typically fragmented, and less than 60% of those are identified [50]. While DDA mode is well-suited for proteomics studies, since protein identification only requires the identification of a minimal number of unique peptides ions that are selected for MS/MS analysis for subsequent database search. However, this acquisition mode tends to be best suited for the identification of high-abundant protein covalent adducts. Therefore, two main data acquisition strategies have been applied to increase the chances of identifying low-abundant protein adducts, following digestion to peptides/amino acids or Edman degradation: 1) using multiple reaction monitoring (MRM) to target specific parent and fragments ions); or 2) forcing all ions that fall within a specified mass range to undergo MS/MS analysis, by data independent analysis (DIA).

MRM acquisition mode is a highly sensitive method, typically performed in triple-quadrupole instruments, where predefined *m/z* ratios are actively searched, and the subsequent fragmentation is only performed on the ions that match the search criteria. This strategy can be applied for the search of adducted peptides, amino acids or *N*-terminal Val hydantoins (in the Hb *N*-alkyl-Edman approach). Parent ion selection is based on the amino acid, peptide or hydantoin with a mass increment of the covalent conjugate. Park’s group used the protein digestion to peptides together with an MRM MS analysis workflow for the in vivo identification of multiple HSA peptides covalently modified with reactive metabolites of drugs such as β-Lactam antibiotics [51], diclofenac [11] nevirapine [52] and abacavir [53] metabolites. Methodologies for the absolute quantitation of specific peptides and Lys HSA adducts formed with acetaminophen [54] and aflatoxin B1 [24], respectively, were also developed based on the MRM acquisition mode. The MRM analysis of adducts obtained upon HSA pronase E digestion to aminoacids was used by Sabbioni et al. [55] for the identification of Lys adducts of 4,4′-methylenediphenyl diisocyanate (MDI) in workers with and without diisocyanate occupational asthma, which enabled the identification of substantially higher levels of adducts in asthma case suggesting that HSA MDI-Lys adducts are suitable for industrial biomonitoring. Selected reaction monitoring (SRM), in which a specific parent ion is selected for subsequent targeted fragmentation reaction, was also used for the targeted identification of specific protein covalent modifications formed upon bioactivation of anti-HIV drugs [36,38].

Untargeted approaches for the identification of multiple covalent conjugates in the Cys34 of HSA and *N*-terminal Val of Hb were also proposed based on MRM. Aldine et al. [56] developed an MRM-based methodology for the identification of multiple adducts formed between reactive carbonyl species and the HSA peptide LQQCPF (obtained upon trypsin and chymotrypsin digestion), that contains Cys34. This was subsequently applied for the identification of HSA Cys34 adducted by acrolein as a marker of oxidative stress in ischemia reperfusion injury during hepatectomy [57]. The untargeted identification of multiple covalent conjugates formed with the *N*-terminal Val of Hb, following *N*-alkyl Edman degradation with FITC, was initially uncovered using MRM acquisition mode [43]. Recently, the use of HRMS DIA was proposed for this untargeted approach [58] as an advantageous acquisition mode for the identification of low-abundant covalent adducts. In fact, DIA strategies [59], emerged to overcome DDA reproducibility and sensitivity drawbacks, by systematically isolating and fragmenting ions on the basis of their *m/z*, and not on their intensities. This acquisition strategy has also shown its utility for the identification and quantification of acetaminophen-derived protein covalent adducts, in three-dimensional human liver microtissues treated with this drug [60]. A DIA-based MS analysis was also proposed by Porter and Bereman [61] for the identification of adducts formed at targeted residues without prior knowledge of the modification. However, the major challenge posed by this acquisition mode is still the data analysis step.

## 4. MS Data Processing and Protein Covalent Adducts Identification

Despite the extensive development of MS data processing platforms for proteomics studies, the data processing step is still a major limitation for the identification of low abundant covalent protein adducts.

Identification of covalent adducts candidates from MRM data rely on the observation of diagnostic fragments belonging to the non-modified peptide, amino acid or the *N*-alkyl Edman hydantoin in the MS/MS data [27,29]. As discussed in the previous sections, this methodology has proved its application for the identification of multiple covalent conjugates formed in targeted residues of proteins. However, it is impractical for unrevealing multiple protein targets of adduction by electrophiles.

The use of HRMS MS1 filtering for the identification of *m/z* values of adducted peptides was referenced in one particular *in vitro* study, concerning the detection of acetaminophen-adducted microsomal proteins upon background subtraction of the isotopically labelled from the non-labelled acetaminophen incubation hydrolysates [62]. However, the application of background filtering strategies for the identification of covalent adducts formed in vivo is anticipated to be difficult due to the diversity and complexity of human matrices.

Proteomics search engines such as Mascot [63], Global Proteome Machine interface (GPM Fury) [64], X!Tandem [65] and Andromeda [66] are traditionally used for the identification of covalent adducts analyzed by DDA mode. These methods consist on matching experimental MS/MS spectra against theoretical *in silico* spectra from a protein database, upon introduction of the (known) mass increment of the covalent modification. These approaches require the availability of a good quality MS/MS spectra of adducted peptides and the prior knowledge of the mass of the modification (“restrictive approaches”). This means that they are only effective if you know what you are looking for. For unknown modifications, they are useless. Unrestrictive or open mass search approaches were developed to overcome this limitation, which use: i) sequence tags to identify the nonmodified peptide in a database and then identify the modification based on the mass difference between the identified and observed peptide (e.g., SPIDER [67]); or ii) spectral alignment with wide tolerant mass range to match all potential peptides in a database with the modified MS/MS spectra (e.g., MSFragger [68], PTMap [69]). The use of data mining algorithms for open modification searches of MS/MS data, which do not require prior knowledge of mass increment of covalent conjugate, were also proposed for the untargeted identification of post translational modifications [70]. 

These methods have the advantage of not needing a list of predefined modifications. However, are depend on databases and their performance depends on the availability of quality MS/MS spectra of adducted peptides. Moreover, these database-dependent methods are usually time-consuming when increasing the number of protein modifications and they report a high rate of false positives.

To overcome the limitations of database-dependent methods, several database-independent algorithms such as DeltAMT (Delta Accurate Mass and Time) [71] and ModifiComb [72] were developed for the detection of post translational modifications of proteins, based on the ∆M of adducted and non-adducted peptides. These methods have the advantage of not depending on databases and do not require prior knowledge of mass increment of covalent conjugate. Thus, although these algorithms are best suited for the identification of high-abundant modifications, they present a potential tool for the identification of covalent adducts formed with unknown exogenous or endogenous electrophiles. 

DIA emerged in the last years to overcome the DDA inability for the detection of low-abundant adducts and, consequently, several data analysis tools were developed for the identification of covalent adducts using DIA data. For instance, a three-step procedure, named Multiplex Adduct Peptide Profiling (MAPP), was developed by Porter et al. [61] for the identification of site specific modifications of targeted peptides that relies on: 1) identification of fragment ion tag which consists of the *b* and *y* ion series also present in the non-adducted peptide; 2) MS1 features are matched to the fragment ion tag; and 3) modified peptides are finally identified upon comparison of modified fragment ions with the unmodified fragment ions to verify the mass increment calculated in the previous step. Egertson et al. [73] proposed the use of Skyline for the proteome wide peptide-identification using DIA data, in which a spectral library is generated using DDA, and chromatograms are extracted from the DIA data for all peptides in the library. However, this methodology can only be applied for the identification of protein adducts that were identified by DDA. If the MS/MS spectrum of adducted peptide is absent from the DDA-generated library it will elude detection. A distinct approach was used by Nesvizhskii’s group for the identification of covalent modifications from DIA [74]. This group developed the open-source software, DIA-UMPIRE, that relies on the detection of peptide and fragment ion features and uses peak elution similarities to group detected fragment and precursor signals. The grouped signals are then assembled into pseudo MS/MS spectra that can be subsequently searched with any analysis tools developed for DDA data, including MS/MS database search engines (e.g., X! Tandem, Mascot, MaxQuant). While DIA-UMPIRE appears as a promising tool for the identification of known protein covalent adducts from DIA data, taking into consideration that DIA-UMPIRE-generated pseudo-spectra are searched by conventional database searching pipelines designed for DDA, this open-source software is not suitable for the identification of unknown modifications. Additionally, pseudo spectra with poor precursor signal are less likely to yield confident identifications, which might compromise the identification of the corresponding adducts. To overcome this limitation, PECAN (PEptide Centric ANalysis) that detects peptides directly from DIA data without prerequisite spectral or retention time libraries, was developed recently [75] as an alternative tool to spectrum-centric tools such and DIA-UMPIRE. However, to our knowledge, its application for the identification of protein covalent adducts is yet to be provided.

Novel methodologies focused on adductomics endpoints are being developed. For instance, our group, inspired by the metabolomics workflow [76], has developed a novel strategy that consists on LC-MS data preprocessing followed by statistical analysis [77]. This strategy was validated using experimental LC-MS data of histones isolated from HepG2 and THLE2 cells exposed to the chemical carcinogen glycidamide. MS1 data was first preprocessed and potential adducts were selected based on the *m/z* increments corresponding to glycidamide incorporation. Then, statistical analysis was applied to identify covalent adducts, corresponding to those ions differently present in cells exposed and not exposed to glycidamide.

## 5. Conclusions

The task of identifying protein covalent adducts is often described as hunting for a “needle in a haystack”. In fact, despite the huge technological advances in MS instrumentation and bioinformatics tools for proteomics studies, enabling the identification of several thousands of proteins in a single injection analysis, these methodologies have very limited success in the identification of low abundant covalent protein adducts. Therefore, MS-based protein targeted or untargeted adductomics studies are only successfully accomplished upon the integrative use of effective and suitable sample pre-treatment, MS analysis and MS data processing procedures. As it was summarized in this review, multiple choices are available for each of these steps and should be judiciously selected according to the aim of the study and the nature of the covalent conjugates. 

Very effective untargeted adductomics MS-based pipelines have already been developed. However, while these untargeted strategies are effective for the identification of adducts in specific (hot-spot) residues of adduction on model proteins, which are advantageous for exposure studies, they are ineffective in providing information on the underlying mechanisms of the chemically-induced adverse effects. Such investigations demand knowing specifically which targeted proteins have a toxicological role, which residues on the protein were modified and the exact chemical structure of the conjugate. There is clearly an opportunity to develop new analytical approaches focused on these endpoints, which will have a huge impact in all fields of adductomics application.

## Abbreviations

BChE	butyrylcholinesterase
DDA	data dependent analysis
DIA	data independent analysis
FITC	fluorescein isothiocyanate
Hb	hemoglobin
HRMS	high resolution mass spectrometry
HAS	Human serum albumin
LDEs	lipid-derived electrophiles
MDI	4,4′-methylenediphenyl diisocyanate
MRM	multiple reaction monitoring
SRM	selected reaction monitoring
